# Environmental sex reversal in parrotfish does not cause differences in the structure of their gut microbial communities

**DOI:** 10.1186/s12866-024-03698-3

**Published:** 2024-12-19

**Authors:** Jingcheng Dai, Teng Wang, Shunda Qiu, Xiaoxue Qi, Juntao Zeng, Changcui Chen, Siqi Wu, Dongru Qiu, Shijie Bai

**Affiliations:** 1 School of Life Science and Technology, Wuhan Polytechnic University, Wuhan, 430023 China; 2 Institute of Deep-sea Science and Engineering, Chinese Academy of Sciences, Sanya, 572000 China; 3 Scientific Observation and Research Station of Xisha Island Reef Fishery Ecosystem of Hainan Province, Sanya Tropical Fisheries Research Institute, Sanya, 572018 China; 4 University of Chinese Academy of Sciences, Beijing, 100049 China; 5 Institute of Hydrobiology, Chinese Academy of Sciences, Wuhan, 430023 China

**Keywords:** Parrotfish, Sex reversal, Gut microflora, 16S rRNA gene sequence, Microbial communities

## Abstract

Parrotfish are a common fish in coral reef areas, but little is known about their gut microbial communities. In addition, parrotfish are capable of sex reversal, usually some males are sexually reversed from females, and it is still not known whether this sex reversal leads to significant changes in gut microbial communities. In this study, we investigated the gut microbial communities of three species of parrotfish including *Scarus forsteni* (4 females and 4 sex-reversed males), *Scarus ghobban* (5 females and 5 sex-reversed males), and *Hipposcarus longiceps* (5 females and 5 sex-reversed males) by using high-throughput sequencing technology. The gut microbial communities of these three species were mainly composed of Pseudomonadota (class Gammaproteobacteria) and Bacillota, while at the family level, they mainly included *Vibrionaceae*, *Burkholderiaceae*, *Enterobacteriaceae*, *Streptococcacea*, and *Erwiniaceae*. Although at the genus level, there were a large number of unclassified lineages, the remaining gut microorganisms were mainly composed of *Vibrio*, *Photobacterium*, *Enterococcus* and *Lactococcus*. Furthermore, we did not find significant differences in gut microbial community structure between the female parrotfish and corresponding female reversed males within each species, even in terms of the structure of gut microbial functional information obtained from 16 S rRNA gene sequence predictions. However, the gut microbial communities of these three species of parrotfish differed significantly not only in their community structure but also in their microbial functional information structure, mainly in terms of aspartate and asparagine biosynthesis, histidine degradation, inositol degradation, heptose biosynthesis, chitin derivatives degradation, enterobactin biosynthesis, and thiazole biosynthesis. Our study provides essential gut microbial community data for understanding the physiology and sex reversal phenomenon in parrotfish.

**Supplementary Information:**

The online version contains supplementary material available at 10.1186/s12866-024-03698-3.

## Introduction

Environmental sex reversal (ESR) is the change of the sex of an individual from male to female or female to male at a specific time in the environment. Creatures with sex reversal mainly have both male and female reproductive organs in their bodies, usually expressing only one, and at a later point the other previously suppressed organ will develop and mature to show the other sex [1, 2]. Sex reversal in fish is well documented in numerous species, and is a complex process with many different types, yet the detailed mechanisms of this process are still poorly understood.

Traditionally, hermaphroditic fish have been divided into those that use genetic sex determination (GSD) and those that use environmental sex determination (ESD), in which environmental factors trigger sexual differentiation, such as temperature sex determination (TSD) [3]. However, these two categories are not one or the other, and under stable environmental conditions, specific pairings of sex chromosomes can consistently result in development of either sex. However, in some extreme environments with sudden changes, such as naturally occurring temperature changes or exogenous drugs or contaminants of anthropogenic origin, other factors may become influential in determining sex phenotypes [4]. In a process also known as environmental sex reversal (ESR), such abrupt environmental changes can interrupt the development of fish species with different sex-determining chromosomes, which may result in individual fish having genetic sex opposite to the most common phenotypic sex [5]. Sex-reversed fish can produce offspring with a disproportionate sex ratio because although their sex phenotype is altered, their genotype does not change, and thus ESR can greatly affect wild fish populations [6]. There have even been suggestions that when trying to artificially manipulate the structure of wild fish populations for a given environment, sex-reversed individuals could be purposefully released to affect the male to female ratio of fish in that environment [7–9]. In addition to this, sex reversal and behavioral differences between wild-type fish may be important for assessing the effects of ESR on fish populations [6]. Adult gynogenetic crucian carp (*Carassius auratus langsdorfii*) can exhibit male spawning behavior when implanted with 11-ketotestosterone [10]. 17alpha-methyltestosterone was used to induce the production of male fighting fish (*Betta splendens*), and the sex-reversed males grew significantly more slowly compared to normal males, while the sex-reversed males showed a decrease in sperm count and viability, resulting in their low reproductive ability [11].

Based on the lengthy evolutionary journey of microorganism, certain bacteria remained as independent, solitary cells, while others formed symbiotic relationships with hosts, inhabiting the digestive tracts of animals, co-adapting with their hosts, and performing crucial functions in the host’s physiology [12]. The coordination between the microorganisms in the gut and the cells of the host is crucial for maintaining the health, survival, and normal functioning [13–15]. The majority of microbes live in the gut and can impact the body’s immune system, helping with nutrient absorption and protecting against infections [16, 17]. Differences in the structure of host-associated gut microbial communities are determined by environmental and host genetic factors [18–20]. Moreover, gut microbes correlate with different host phenotypes [21], and sex differences have a significant impact on their physiology and behavior [22], hence some studies have found differences in the gut microbes of females and males of the same species, including fish, mice, and humans [23–25]. Interestingly, in an experiment with mice, males and females had different gut microbes, but when the males were castrated, their gut flora changed, suggesting that sex hormones can affect the composition of the gut microbial community [26]. Meanwhile, gut microbes can also alter the host’s sex hormone levels, thus affecting the host’s phenotype and resistance to disease [27]. However, the correlation between the phenomenon of sex reversal in fish and their gut microbes is still unknown to us.

The labrid fishes (Labridae), including wrasse, weed-whiting, and parrotfish, are a species group of marine perciform that occupy different marine habitats around the world [28–30]. Sequential hermaphroditism, in which individuals change sex as a normal part of their life history, is widespread among fishes. Typically, protogynous sex change from female to male in fishes involves remarkable and apparently irreversible changes in gonadal anatomy and function, as well as in coloration and behavior [31]. The family Labridae includes about 600 species of fish that live in environments including shallow tropical coral reefs, seagrass beds, and temperate rocky reefs [28, 29]. Parrotfishes are a monophyletic group of 10 genera and around 100 species. They live in tropical and subtropical seas, with the greatest species diversity in the Indo-Pacific region. They live almost exclusively in coral reef environments and are characteristic of coral reef fish populations worldwide [32, 33]. The Pacific longnose parrotfish *Hipposcarus longiceps*, is an important component of commercial and subsistence reef fisheries throughout the Indo-Pacific region and is one of the highest-yielding reef fishes in the Pacific Islands region. Moreover, *H. longiceps* is a medium to large bodied parrotfish species, with fork lengths that can reach around 60 cm. They often form large schools to feed and breed, roaming back and forth in coral reef environments. Also, *H. longiceps* are thought to play an important ecological role on reefs, acting as scrapers and mini-diggers, reducing turf and macroalgal cover, and producing bare substrate through their predatory behaviour [34, 35]. The blue-barred parrotfish *Scarus ghobban*, is a widespread species in the Indo-Pacific region, ranging from the Red Sea to the tropical eastern Pacific, but its genetic diversity is relatively low, clustered into two main clades, the Pacific and Indian Ocean clades [36]. Forsten’s parrotfish *Scarus forsteni*, is widely distributed in coral reef environments, found mainly in exposed reef areas [37]. Parrotfish have both positive and negative impacts on coral reef ecosystems. On the one hand, they help control the abundance of macroalgae which can outcompete corals. On the other hand, they directly prey on corals, leading to decreased coral abundance, diversity, and colony size. Parrotfish can also promote coral growth by dispersing coral fragments and creating space for coral settlement, but they can also cause damage to adult corals and mobilize significant amounts of carbonate sediment, which can create stressful conditions for the coral. Therefore, it is crucial to consider both the positive and negative impacts of parrotfish on the coral reef ecosystem [38–40]. Further research is needed to understand the role of parrotfish in maintaining the balance of the ecosystem and to develop sustainable management practices that minimize the negative impacts of these fish while maximizing their positive ones.

To date, there have been a few of studies reported on the gut microbes of parrotfish [41–44], and some researchers found that the coral-macroalgal shift did not influence the diversity, richness or variability of fish gut bacteriomes following climate-driven regime shifts. Additionally, some results showed the connectivity of a shared microbiome between seagrass, fish and sediments, and that bacteria cell abundances from feces and distal gut contents ranged two orders of magnitude among the different coral reef fish species. But there have been relatively few comparative studies on the gut microbes of females and males after sex reversal in parrotfish. Here, we collected different sexes of *S. forsteni* and *H. longiceps* from the coral reef areas of East Island and Yongle Island in the Xisha Islands, China, respectively, and we also collected different sexes of *S. ghobban* from the coral reef area in Dongsha Island, China. We identified their environmental sex reversal phenomenon and then studied their gut microorganisms. We aimed to address the following topics: (1) To reveal the structure and composition of gut microbial communities in three species of parrotfish, *H. longiceps*, *S. ghobban* and *S. forsteni*; (2) To compare the structure of gut microbial community in three species of parrotfish with environmental sex reversal, and to investigate whether there is a correlation between environmental sex reversal and gut microbial community structure.

## Materials and methods

### Sample collection

**Fig. 1:**
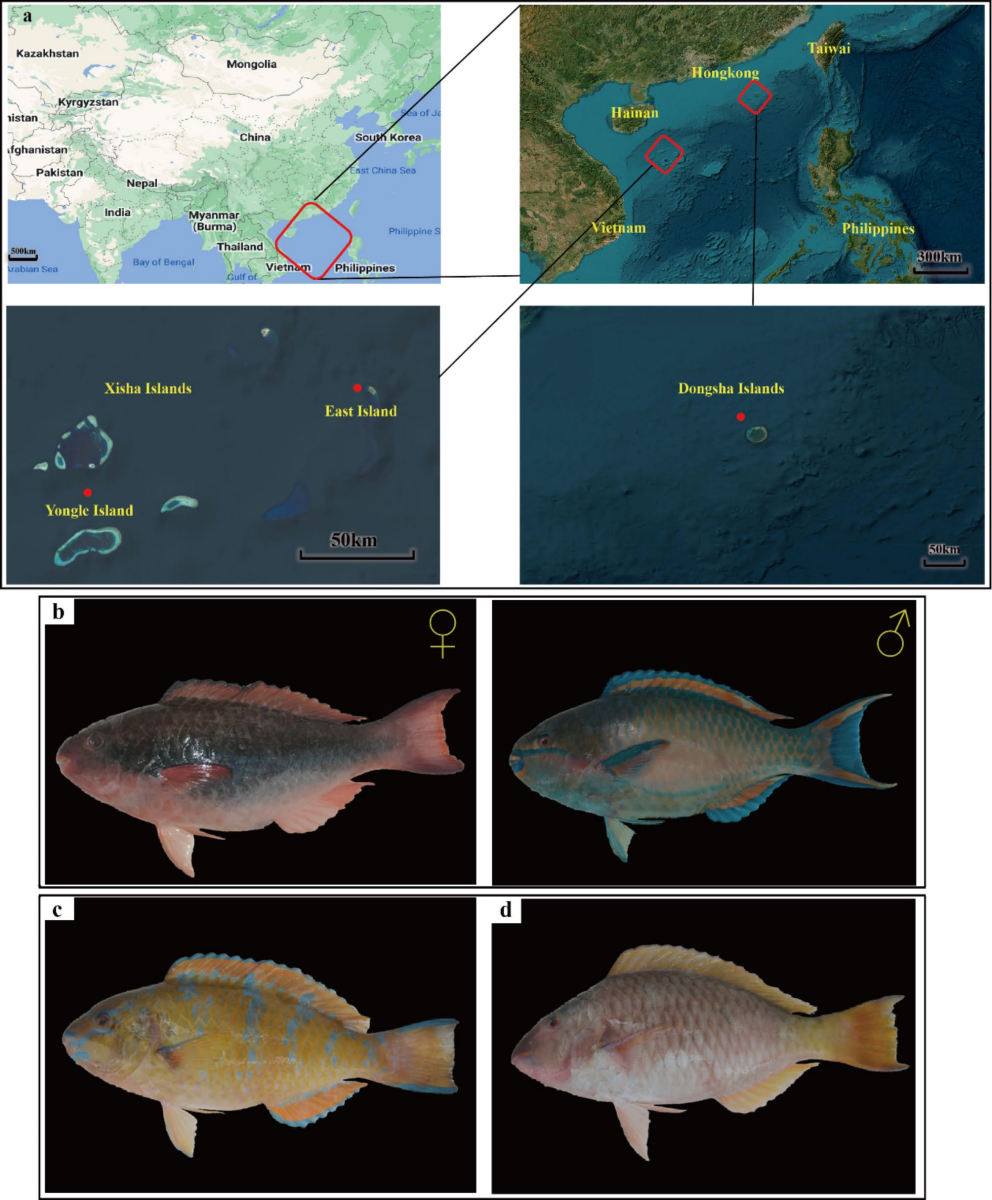
Information and photographs of sampling sites for the three species of parrotfish. The three sampling sites are as follows (**a**): East Island, Yongle Island, Dongsha Islands. In total, we collected three species of parrotfish: female (left ♀) and male (right ♂) Forsten’s parrotfish (*Scarus forsteni)* from East Island (**b**), female and male Blue-barred parrotfish (*Scarus ghobban*) from Dongsha Islands (**c**), and female and male Longnose parrotfish (*Hipposcarus longiceps*) from Yongle Island (**d**)

During the period from April 28 to May 14, 2022, we conducted a sea survey of the fishery resources of the Xisha Islands and the Dongsha Islands in China, and within this time-period, we collected 4 female *Scarus forsteni* (Forsten’s parrotfish) and 4 sex-reversed male *S. forsteni* in the coral reef area of the East Island in the Xisha Islands, 5 female *Hipposcarus longiceps* (pacific longnose parrotfish) in the coral reef area of Yongle Island in the Xisha Islands and 5 sex-reversed male *H. longiceps*, and finally we collected 5 female *Scarus ghobban* (blue-barred parrotfish) and 5 sex-reversed male *S. ghobban* in the coral reef area of the Dongsha Islands. The sampling location information, temperature, salinity and photographs of the three species of parrotfish are shown in the supplemental table S1 and Fig. 1. After being anesthetized with MS-222 at a dose of one thousandth of the weight of the fish, all fish were dissected under sterile conditions. Approximately 1.5–2 cm of intestinal tissue was cut and collected in a 1.5 mL sterile microcentrifuge tube. These intestinal samples were then stored in the refrigerator at -80℃ for further analysis. The secondary sexual characteristics of fish were determined by direct visual inspection of the male and female gonads. In cases where it was still impossible to distinguish, gonadal tissue maturity was determined by examination under a dissection microscope [45].

### Extraction and sequencing of gut microbial DNA

To extract gut microbial DNA, the following steps were taken: (1) A mixture of 1000 ul of CTAB lysate and 20 ul of lysozyme was prepared and the appropriate sample was added. The mixture was then incubated in a water bath at 65 °C for 2 h, followed by inversion and mixing to promote lysis. (2) The resulting supernatant was centrifuged, and then an equal volume of phenol (pH 8.0): chloroform: isoamyl alcohol (25:24:1) was added. The mixture was inverted and centrifuged at 12,000 rpm for 10 min. (3) An equal volume of chloroform: isoamyl alcohol (24:1) was added to the supernatant, which was then inverted, mixed, and centrifuged at 12,000 rpm for 10 min. (4) The supernatant was transferred to a 1.5 mL EP tube, and 75% volume of isopropanol was added. The mixture was shaken and allowed to precipitate at -20 degrees for two hours. (5) The samples were then centrifuged at 12,000 rpm for 10 min and the supernatant decanted, taking care not to pour out the precipitate. The pellet was washed twice with 1 ml of 75% ethanol. The remaining small amount of liquid was collected by centrifugation again and then pipetted out. (6) The samples were dried at room temperature on an ultra clean bench. Finally, 51µL ddH_2_O was added to dissolve the DNA sample. If necessary, the sample was incubated at 55 to 60ºC for 10 min to facilitate dissolution.

To quantify the extracted DNA, a Qubit fluorometer (Invitrogen Inc. Manufacturer: Life Technologies Holdings Pte Ltd, Singapore) was used. The V3-V4 region of the 16 S rRNA gene was amplified using the primer pair 341 F (5′-CCTAYGGGRBGCASCAG-3′) and 806R (5′- GGACTACNNGGGTATCTAAT-3′) in triplicate, as per Xiong et al. (2015). The PCR cycling conditions were as follows: denaturation at 95 °C for 5 min, followed by 34 cycles at 94 ℃ for 60 s, 57 °C for 45 s, 72 °C for 60 s, and a final extension at 72 °C for 10 min. The TaKaRa purification kit (TaKaRa, Japan) was used for purification of the products of the triplicate PCR reactions, which were then combined. The TruSeq DNA sample preparation kit (Illumina, San Diego, CA, USA) was used for library construction as per the manufacturer’s instructions. Novogene Co. Ltd. (Beijing, China) sequenced the libraries on the Novaseq6000 platform (Illumina) with a paired-end 250 bp sequence reads.

### Microbial data processing and statistical analysis

After completing the sequencing and obtaining the raw data, sequences were sorted to individual samples according to barcodes, allowing for one mismatch, after which the barcodes, as well as forward and reverse primer sequences, were removed from the sequences to obtain clean data. We used FLASH (version 1.2.8) [46] to obtain paired-end full-length sequences of sufficient length, with at least 30 bp of overlap. We then used Btrim (version 0.2.0) to select high quality sequences without Ns and between 400 bp and 430 bp in length for subsequent analyses [47]. UNOISE3 was used to generate amplicon sequence variants (ASVs) with default settings [48] without singletons, and subsequently a representative sequence from each ASV was selected for taxonomic annotation, and taxonomic information was obtained using the RDP classifier for comparison with the SILVA 138 database including bacterial, archaeal and eukaryotic sequences [49]. The generated ASV table was used in the subsequent analyses. The diversity of microbial communities in different parrotfish gut samples was determined by statistical analysis of α-diversity indices, based on different groups, such as species and sex, to compare whether there was a significant difference in the α-diversity indices between different groups. The Chao1 index, Shannon and Pielou evenness indices were calculated using the vegan package in R language version 4.0 [50]. The indicator ASVs of microbial communities were classified using IndVal.g analysis of the R package labdsv tool [51]. Only ASVs with highly significant indicator values (IndVal.g index > 0.95, *p* < 0.001) were considered strict habitat specialists [52]. To investigate the differences in the gut microbial community structure of different species of parrotfish, and to investigate whether there were differences between female and male (after the occurrence of environmental sex reversal) in the gut microbial community structure of the three different parrotfish species, non-metric multidimensional scaling (NMDS) method, a statistical tool based on ß-diversity, was used to calculate Bray-Curtis and Jaccard distance matrices. Both the Bray-Curtis and Jaccard distance matrices are commonly used ecological metrics to measure similarity or difference between samples, and they differ from each other in whether or not data on the relative abundance of species in the sample are considered to calculate the degree of difference in community compositional structure. We also tested whether there were any dissimilarities between the defined groupings (including *H. longiceps*, *S. ghobban* and *S. forsteni*, and also females and males of each species of parrotfish) by performing permutational multivariate analysis of variance (PERMANOVA), multi response permutation procedure (MRPP), and a one-way ordered analysis of similarity (ANOSIM). The use of PERMANOVA allows for the analysis of the degree to which the different grouping factors explain the differences in the samples and the use of the permutation test for significance statistics, MRPP is a statistical method used to analyse the similarity between groups of high-dimensional data, which is based on distance matrices and is used to determine whether the data between the groups are significantly different or not, and ANOSIM is a non-parametric test used to test the significance of the differences between the groups. Data analysis followed the method previously described by Bai and Hou [53]. Comparisons of α-diversity between different groups, for instance, significant differences between species or sexes, were made using the Wilcoxon rank sum test of IBM SPSS Statistics 19.

### Comparison of potential functions of intestinal microbial communities in Parrotfish

To obtain potential functional information on the gut microbial communities of the three different parrotfish species, we used PICRUSt version 2 software to predict the potential functions of the microbial communities for all gut samples based on three databases, including the MetaCyc, which contains pathways involved in both primary and secondary metabolism, as well as associated metabolites, reactions, enzymes, and genes [54], the Clusters of Orthologous Genes (COG), also known as Clusters of Orthologous Groups of proteins, has been a popular tool for functional and comparative genomics of bacteria and archaea [55], and the Kyoto Encyclopedia of Genes and Genomes (KEGG), which is a database resource for understanding high-level functions and utilities of the biological system, such as the cell, the organism and the ecosystem, from molecular-level information [56]. In simple terms, the representative sequences were first clustered and aligned, and then dropped into the reference phylogeny based on the EPA-NG method. The gene family abundance and gene copy number were predicted by the hidden state prediction with default parameters. After that, the ASV table abundance was normalized by the number of predicted genes, and finally the predicted functional profile was determined for each sample based on different databases [57].

After obtaining information on the potential functions of these gut microbial communities, we constructed functional and sample-coupled datasets and performed NMDS analysis based on Bray-Curtis and Jaccard distance matrices. Meanwhile, we analyzed the similarities and differences in the potential functional community structure of the parrotfish gut microbes in different grouping cases using three statistical methods, namely PERMANOVA, MRPP and ANOSIM. In addition, we used the STAMP software to present significant functional differences, STAMP is a graphical software package that provides statistical hypothesis testing and exploratory plots for analysing taxonomic and functional characteristics, and it supports tests that compare paired samples or samples divided into two or more treatment groups, while the software provides effect sizes and confidence intervals to allow for a rigorous assessment of the biological relevance of test results [58].

## Results

### Sequencing statistics and microbial diversity

**Fig. 2:**
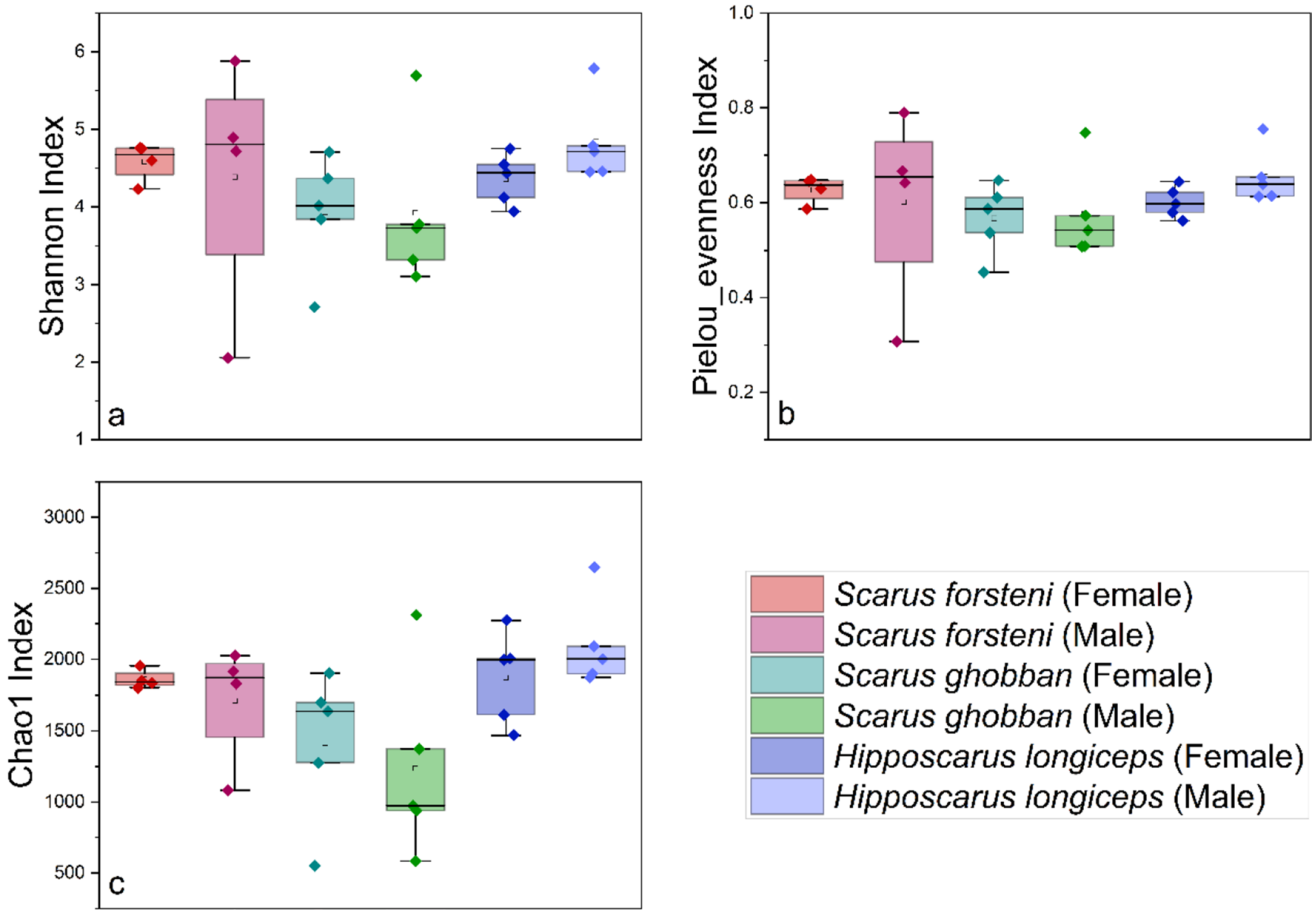
Comparisons of four α-diversity indices. Shannon index (**a**),Pielou evenness index (**b**), and Chao1 index (**c**), of the 28 parrotfish gut samples. We also calculated and compared these three α-diversity indices in the gut microbial communities of females and males from *S. forsteni*, *S. ghobban*, and *H. longiceps*, respectively, the male samples here refer to males after sexual reversal. The significance of the differences was determined using the Wilcoxon rank-sum test. A single asterisk (*) indicates a significant difference at the 0.05 level, a double asterisk (**) indicates a significant difference at the 0.01 level. These results were obtained from the ASVs datasets

**Fig. 3:**
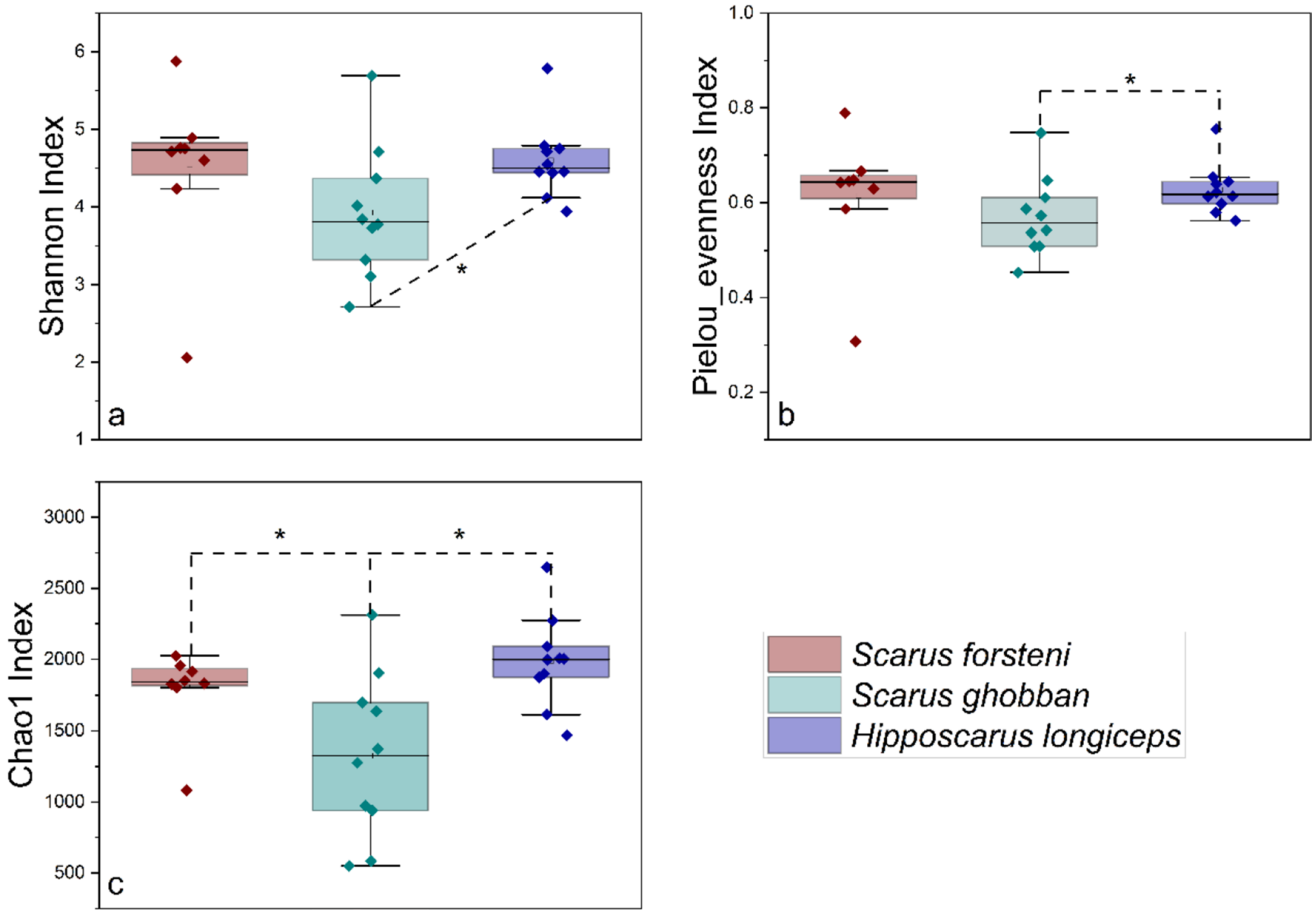
Comparisons of four α-diversity indices, Shannon index (**a**), Pielou evenness index (**b**), and Chao1 index (**c**), of the 28 gut specimens from *S. forsteni*, *S. ghobban*, and *H. longiceps*. The significance of the differences was determined using the Wilcoxon rank-sum test. A single asterisk (*) indicates a significant difference at the 0.05 level, a double asterisk (**) indicates a significant difference at the 0.01 level. These results were obtained from the ASVs datasets

After conducting a quality assessment, a total of 1,407,999 sequences were obtained from 28 gut samples of parrotfish, the parrotfish *S. forsteni* included 4 female and 4 male samples, the parrotfish *S. ghobban* included 5 female and 5 male samples, and the parrotfish *H. longiceps* also had 5 female and 5 male samples (Fig. 1). the female and male (sex-reversed) of Forsten’s parrotfish (*Scarus forsteni*) from East Island were determined by direct visual inspection of changes in body colour and the male and female gonads. The female and male Blue-barred parrotfish (*Scarus ghobban*) from Dongsha Islands, and female and male Longnose parrotfish (*Hipposcarus longiceps*) from Yongle Island were determined by gonadal tissue examination under a dissection microscope. An average of 50,286 ± 7,826 sequences were obtained for each sample. To obtain a more accurate α-diversity results, we rarefied the sequences to 30,079 for each sample, and then used this set of sequences to analyze the microbial diversity, composition, and structure. The α-diversities of microbial communities from the gut of different parrotfish samples were calculated. The Shannon, Pielou evenness, as well as the Chao1 index, indicated that there was no significant difference in gut microbial α-diversity between females and males (with environmental sex reversal) in any of the three species of parrotfish (Fig. 2). In addition, when comparing differences between gut microbial communities of different species of parrotfish alone were considered, ignoring any sexual differences, the microbial α-diversity of gut was significantly higher in *H. longiceps* than in *S. ghobban* based on the results of the Shannon index, and the Chao1 index. Also, the gut microbial α-diversity of *S. forsteni* was significantly higher than in *S. ghobban* based on the results of the Chao1. Although no significant differences were observed, the Pielou evenness index still showed *H. longiceps* and *S. forsteni* to be higher than *S. ghobban*, and *S. forsteni* was also higher than *S. ghobban* in terms of the Shannon index (Fig. 3).

### Gut microbial community structure and composition of the three species of parrotfish

**Fig. 4:**
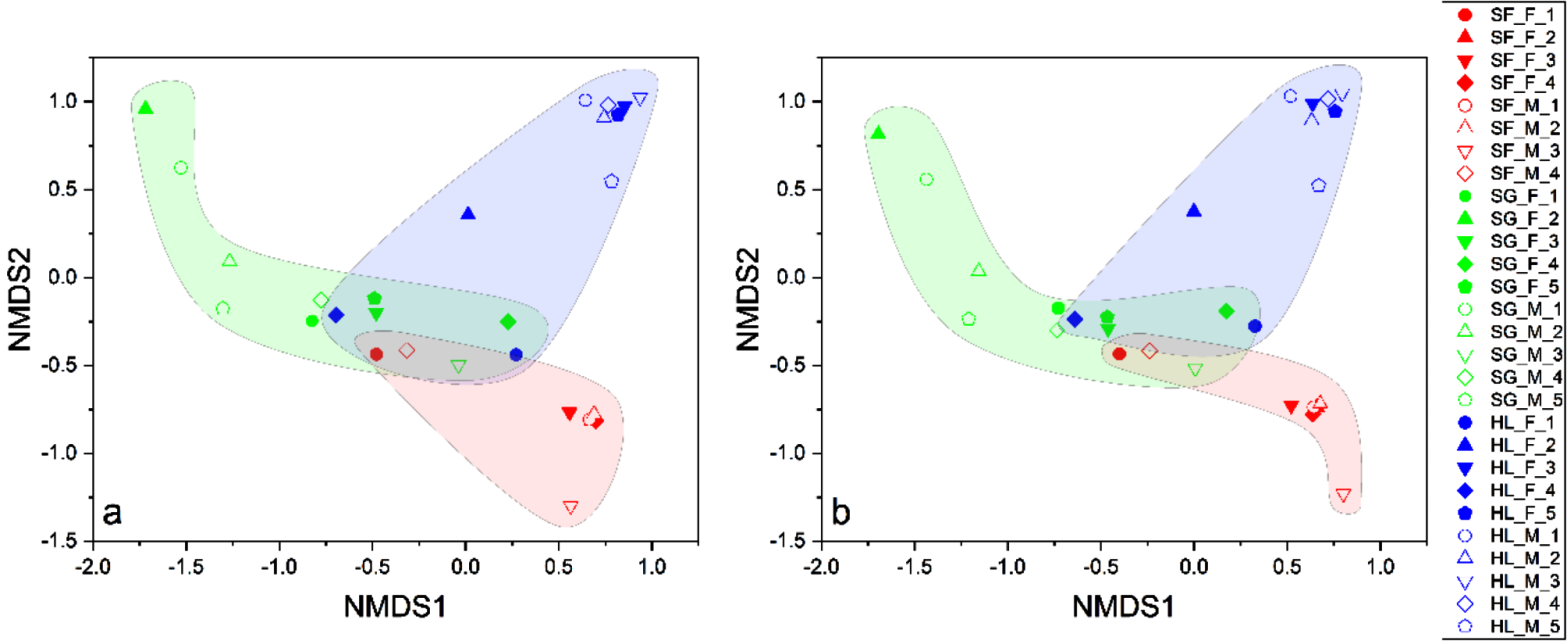
NMDS analysis of the gut microbial communities separated the samples into three principal groups corresponding to the parrotfish species *Scarus forsteni*, *Scarus ghobban*, and *Hipposcarus longiceps*. Results were derived from the ASVs datasets, with the left (stress = 0.07) and right (stress = 0.067) plots calculated using the Bray-Curtis dissimilarity index and Jaccard similarity index, respectively

From the NMDS analysis conducted, the gut microbial communities revealed three main groups - the *S. forsteni* gut microbial community group, *S. ghobban* gut microbial community group, and *H. longiceps* gut microbial community group (Fig. 4). Further statistical analysis, which involved the multi-response permutation procedure (MRPP), one-way ordered analysis of similarity (ANOSIM), and permutational multivariate analysis of variance (PERMANOVA), revealed significant differences in the gut microbial community structure of these three species of parrotfish (Table 1). However, no significant differences were found in gut microbial community structure between male and female parrotfish (following sex reversal) across all three species (Table 2).

**Fig. 5:**
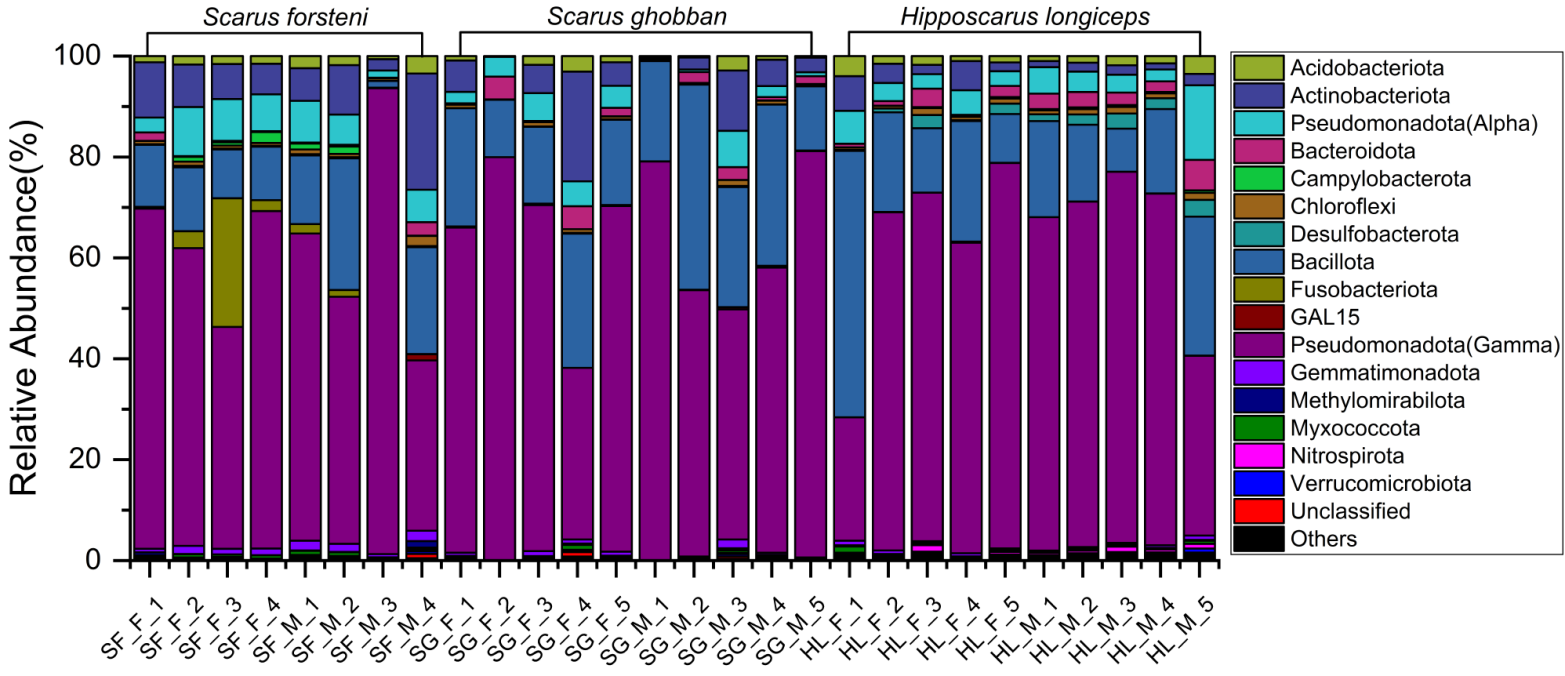
Gut microbial community members of *S. forsteni*, *S. ghobban*, and *H. longiceps* at the phylum level. F refers to female samples and M refers to male following sex reversal samples

The gut microbial communities of three different parrotfish species were analyzed at the phylum, family, and genus levels. The results revealed differences in relative abundances and provided detailed information on their composition (Figs. 5, S1, S2). The dominant bacterial phyla in the gut samples of all three species were Pseudomonadota (class Gammaproteobacteria) and Bacillota. The relative abundances of Pseudomonadota (class Gammaproteobacteria) in the gut of *S. forsteni* ranged from 34 to 92%, in the gut of *S. ghobban* from 34 to 81%, and in the gut of *H. longiceps* from 24 to 76%. Bacillota accounted for 1–21% in the gut of *S. forsteni*, 11–41% in the gut of *S. ghobban*, and 9–53% in the gut of *H. longiceps*. At the family taxonomic level, *Vibrionaceae* and *Burkholderiaceae* (affiliated with the Pseudomonadota), were the dominant bacterial lineages in the *S. forsteni* gut samples. *Enterobacteriacea*e (Pseudomonadota), *Vibrionaceae*, and *Streptococcaceae* (Bacillota) were dominant in the gut samples of *S. ghobban*. Like the *S. ghobban* gut, the gut microbial communities of the *H. longiceps* were also dominated by *Enterobacteriaceae* and *Streptococcaceae*, along with *Erwiniaceae* (Pseudomonadota). Furthermore, at the genus level, the gut microbial communities of *S. forsteni* were mainly composed of *Vibrio*, *Photobacterium*, and *Ralstonia*. The gut microbial compositions of *S. ghobban* and *H. longiceps*, both, contained many unclassified genera, the relative abundance ranges from 17 to 62.92%, with a mean of 36.07%. In addition to these unclassified microbial taxa, *Photobacterium*, *Lactococcus*, and *Enterococcus* were the dominant bacterial genera in the gut samples of *S. ghobban*, while *Enterococcus* and *Lactococcus* were also dominant in the gut samples of *H. longiceps*.

**Table 1:** Tests for dissimilarity in the gut microbial community structure of *Scarus forsteni*, *Scarus ghobban*, and *Hipposcarus longiceps* based on Bray-Curtis and Jaccard distances

Group	Bray-Curtis	Jaccard
	*p*	*p*
Group (*S. forsteni* and *S. ghobban*)		
PERMANOVA	0.001(**), Pseudo-F = 12.101	0.003(**), Pseudo-F = 4.564
MRPP	0.001(**), Delta = 0.549	0.002(**), Delta = 0.646
ANOSIM	0.001(**), *r* = 0.916	0.004(**), *r* = 0.460
Group (*S. forsteni* and *H. longiceps*)		
PERMANOVA	0.001(**), Pseudo-F = 12.333	0.001(**), Pseudo-F = 7.017
MRPP	0.001(**), Delta = 0.589	0.001(**), Delta = 0.608
ANOSIM	0.001(**), *r* = 0.979	0.002(**), *r* = 0.708
Group (*S. ghobban* and *H. longiceps*)		
PERMANOVA	0.001(**), Pseudo-F = 4.990	0.002(**), Pseudo-F = 5.675
MRPP	0.001(**), Delta = 0.559	0.002(**), Delta = 0.661
ANOSIM	0.001(**), *r* = 0.425	0.001(**), *r* = 0.632

* Difference is significant at 0.05 level, ** difference is significant at 0.01 level. *S. forsteni* refer to the forsten’s parrotfish *Scarus forsteni*; *S. ghobban* refer to the blue-barred parrotfish *Scarus ghobban*; and *H. longiceps* refer to the pacific longnose parrotfish *Hipposcarus longiceps*

**Table 2:** Tests for dissimilarity in the gut microbial community structure of *Scarus forsteni*, *Scarus ghobban*, and *Hipposcarus longiceps* based on Bray-Curtis and Jaccard distances

Group	Bray-Curtis	Jaccard
	*p*	*p*
*S. forsteni* (Females and Males after sexual reversal)		
PERMANOVA	0.751, Pseudo-F = 0.610	0.543, Pseudo-F = 0.568
MRPP	0.741, Delta = 0.602	0.508, Delta = 0.604
ANOSIM	0.810, *r* = -0.083	0.666, *r* = -0.125
*S. ghobban* (Females and Males after sexual reversal)		
PERMANOVA	0.905, Pseudo-F = 0.561	0.503, Pseudo-F = 0.908
MRPP	0.837, Delta = 0.537	0.507, Delta = 0.697
ANOSIM	0.849, *r* = -0.092	0.567, *r* = -0.036
*H. longiceps* (Females and Males after sexual reversal)		
PERMANOVA	0.186, Pseudo-F = 1.322	0.124, Pseudo-F = 1.759
MRPP	0.185, Delta = 0.582	0.103, Delta = 0.602
ANOSIM	0.190, *r* = 0.088	0.095, *r* = 0.132
All (Females and Males after sexual reversal)		
PERMANOVA	0.935, Pseudo-F = 0.447	0.517, Pseudo-F = 0.844
MRPP	0.928, Delta = 0.742	0.528, Delta = 0.752
ANOSIM	0.909, *r* = -0.047	0.696, *r* = -0.033

* Difference is significant at 0.05 level, ** difference is significant at 0.01 level. *S. forsteni* refer to the forsten’s parrotfish *Scarus forsteni*; *S. ghobban* refer to the blue-barred parrotfish *Scarus ghobban*; and *H. longiceps* refer to the pacific longnose parrotfish *Hipposcarus longiceps*

### Functional community structure of gut microbial communities from the three species of parrotfish

**Fig. 6:**
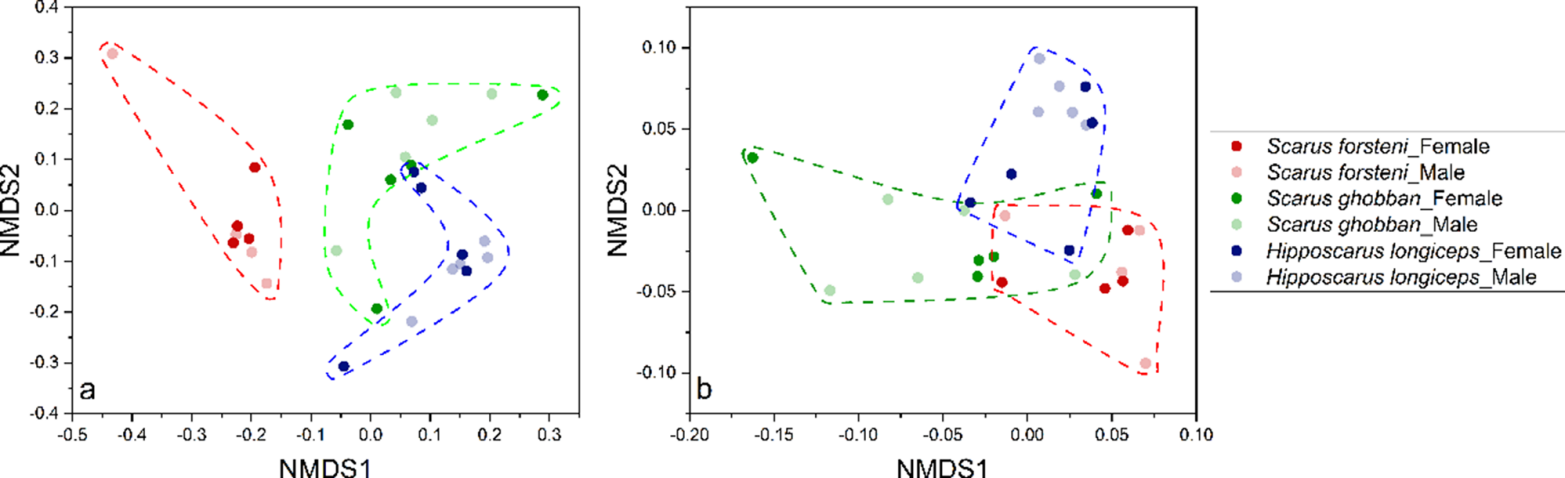
NMDS analysis of the gut microbial functional communities separated the samples into three principal groups, one composed of the gut samples of *Scarus forsteni*, a second group composed of gut samples from the *Scarus ghobban*, and a third group composed of gut samples from *Hipposcarus longiceps*. The results are based on the functional information predicted by PICRUSt2 using the KEGG database. The plots on the left (stress = 0.099) and right (stress = 0.095) were calculated using the Bray-Curtis dissimilarity index (**a**) and the Jaccard similarity index (**b**), respectively

To better investigate the potential effects of differences in the gut microbial community structure of the three parrotfish species we performed functional predictions of the gut microbial community samples using PICRUSt2. We performed functional prediction analyses for each parrotfish species’ gut microbial community based on three different databases, MetaCyc, KEGG and COG. Subsequently, we used the functional information obtained from these predictions to correspond with the respective samples, constructed a functional sample information matrix, and then performed NMDS analysis and compared whether the functions between the groups were statistically different in terms of community structure. The analysis showed that the information on the gut microbial functions of parrotfish generated from the MetaCyc, KEGG, or COG databases formed three distinct groups that were based on parrotfish species (Fig. 6, S3, S4). Further statistical analyses showed significant differences between these different groups, but no significant differences between females and males after sexual reversal (Tables 3 and 4, supplemental table S2 to S5).

**Table 3:** Tests of dissimilarity in the function of gut microbial communities of *Scarus forsteni*, *Scarus ghobban*, and *Hipposcarus longiceps* based on Bray-Curtis and Jaccard distances. The prediction of gut microbial community function was performed by PICRUSt2 based on the KEGG (Kyoto Encyclopedia of Genes and Genomes) database

Group	Bray-Curtis	Jaccard
	*p*	*p*
Group (*S. forsteni* and *S. ghobban*)		
PERMANOVA	0.001(**), Pseudo-F = 9.724	0.001(**), Pseudo-F = 6.191
MRPP	0.001(**), Delta = 0.202	0.002(**), Delta = 0.068
ANOSIM	0.001(**), *r* = 0.469	0.001(**), *r* = 0.341
Group (*S. forsteni* and *H. longiceps*)		
PERMANOVA	0.001(**), Pseudo-F = 15.342	0.001(**), Pseudo-F = 9.334
MRPP	0.001(**), Delta = 0.174	0.001(**), Delta = 0.060
ANOSIM	0.001(**), *r* = 0.716	0.001(**), *r* = 0.721
Group (*S. ghobban* and *H. longiceps*)		
PERMANOVA	0.048(*), Pseudo-F = 2.425	0.001(**), Pseudo-F = 7.550
MRPP	0.013(*), Delta = 0.120	0.001(**), Delta = 0.067
ANOSIM	0.009(**), *r* = 0.148	0.001(**), *r* = 0.524

* Difference is significant at 0.05 level, ** difference is significant at 0.01 level. *S. forsteni* refer to the Forsten’s parrotfish *Scarus forsteni*; *S. ghobban* refer to the blue-barred parrotfish *Scarus ghobban*; and *H. longiceps* refer to the pacific longnose parrotfish *Hipposcarus longiceps*

**Table 4:** Tests of dissimilarity in the function of gut microbial communities of *Scarus forsteni*, *Scarus ghobban*, and *Hipposcarus longiceps* based on Bray-Curtis and Jaccard distances. The prediction of gut microbial community function was performed by PICRUSt2 based on the KEGG (Kyoto Encyclopedia of Genes and Genomes) database

Group	Bray-Curtis	Jaccard
	*p*	*p*
*S. forsteni* (Females and Males after sexual reversal)		
PERMANOVA	1.000, Pseudo-F = 0.403	0.842, Pseudo-F = 0.360
MRPP	0.882, Delta = 0.190	0.768, Delta = 0.063
ANOSIM	0.821, *r* = -0.094	0.655, *r* = -0.115
*S. ghobban* (Females and Males after sexual reversal)		
PERMANOVA	0.888, Pseudo-F = 0.223	0.914, Pseudo-F = 0.308
MRPP	0.872, Delta = 0.227	0.889, Delta = 0.077
ANOSIM	0.916, *r* = -0.140	0.893, *r* = -0.128
*H. longiceps* (Females and Males after sexual reversal)		
PERMANOVA	0.544, Pseudo-F = 0.882	0.074, Pseudo-F = 1.992
MRPP	0.462, Delta = 0.168	0.067, Delta = 0.057
ANOSIM	0.566, *r* = -0.024	0.097, *r* = 0.188
All (Females and Males after sexual reversal)		
PERMANOVA	0.989, Pseudo-F = 0.163	0.827, Pseudo-F = 0.496
MRPP	0.984, Delta = 0.247	0.731, Delta = 0.081
ANOSIM	0.967, *r* = -0.053	0.646, *r* = -0.024

* Difference is significant at 0.05 level, ** difference is significant at 0.01 level. *S. forsteni* refer to the Forsten’s parrotfish *Scarus forsteni*; *S. ghobban* refer to the blue-barred parrotfish *Scarus ghobban*; and *H. longiceps* refer to the pacific longnose parrotfish *Hipposcarus longiceps*

### Screening of different parrotfish gut microbial indicators and demonstration of major functional differences

**Fig. 7:**
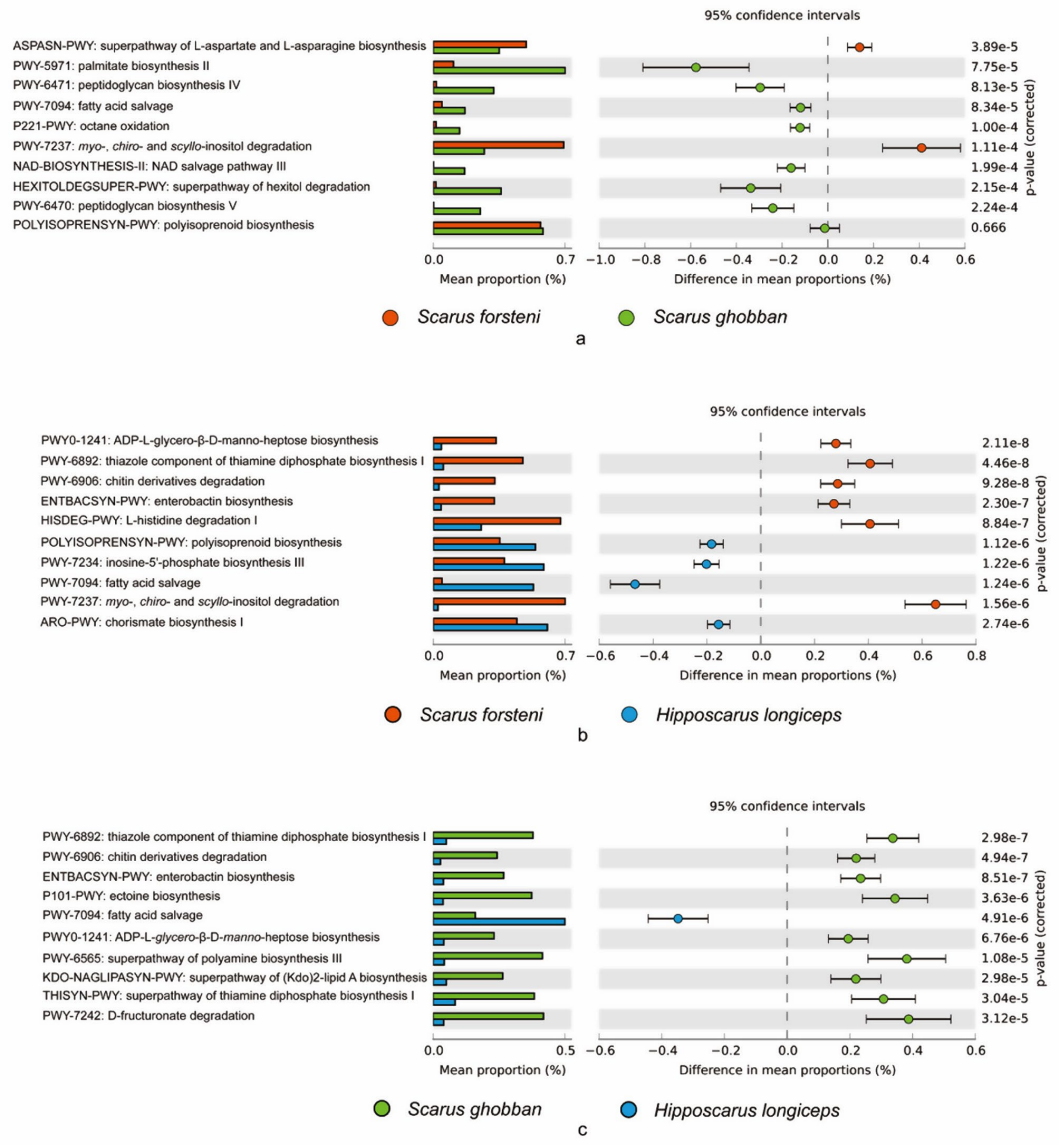
Top 10 differential function information between different species of parrotfish. **a**: *S. forsteni* vs. *S. ghobban*; **b**: *S. forsteni* vs. *H. longiceps*; **c**: *S. ghobban* vs. *H. longiceps*; The functional information was predicted by PICRUSt2 based on the indicator ASVs against MetaCyc database

Structural differences in the gut microbial communities of the three species of parrotfish, *S. forsteni*, *S. ghobban* and *H. longiceps*, were revealed, but the indicators of the different species and the functions they represent still required further analysis and interpretation. Therefore, we explored the detailed differences between different parrotfish gut samples at the ASV level. ASVs with IndVal.g > 0.95, *p* < 0.001 were treated as the indicators of each group. After analysis, 25, 0, and 7 indicator ASVs were found in the gut samples of *S. forsteni*, *S. ghobban*, and *H. longiceps*, respectively. These ASVs were chosen for functional information prediction by PICRUSt2, using the MetaCyc, KEGG, and COG databases. This functional information was analyzed by STAMP to reveal major functional differences between groups. Based on the results of PICRUSt2 against MetaCyc database, the indicator gut microbes of *S. forsteni* were significantly more involved in the superpathway of L-aspartate and L-asparagine biosynthesis and the pathway of myo-, chiro- and scyllo-inositol degradation than in the gut of *S. ghobban* (Fig. 7a). In the comparison of the *S. forsteni* and *H. longiceps* indicator organisms, it was found that the ADP-L-glycero-β-D-manno-heptose biosynthesis, thiazole component of thiamine diphosphate biosynthesis I, chitin derivatives degradation, enterobactin biosynthesis, L-histidine degradation I, and also the myo-, chiro- and scyllo-inositol degradation were enriched in the gut of *S. forsteni* than the *H. longiceps* (Fig. 7b). Meanwhile in the guts of *S. ghobban* and *H. longiceps*, the thiazole component of thiamine diphosphate biosynthesis I, chitin derivatives degradation, enterobactin biosynthesis, ectoine biosynthesis, ADP-L-glycero-β-D-manno-heptose biosynthesis, superpathway of polyamine biosynthesis III, superpathway of (Kdo)2-lipid A biosynthesis, superpathway of thiamine diphosphate biosynthesis I, and D-fructuronate degradation were significantly higher in the gut of *S. ghobban* than in *H. longiceps* (Fig. 7c).

**Fig. 8:**
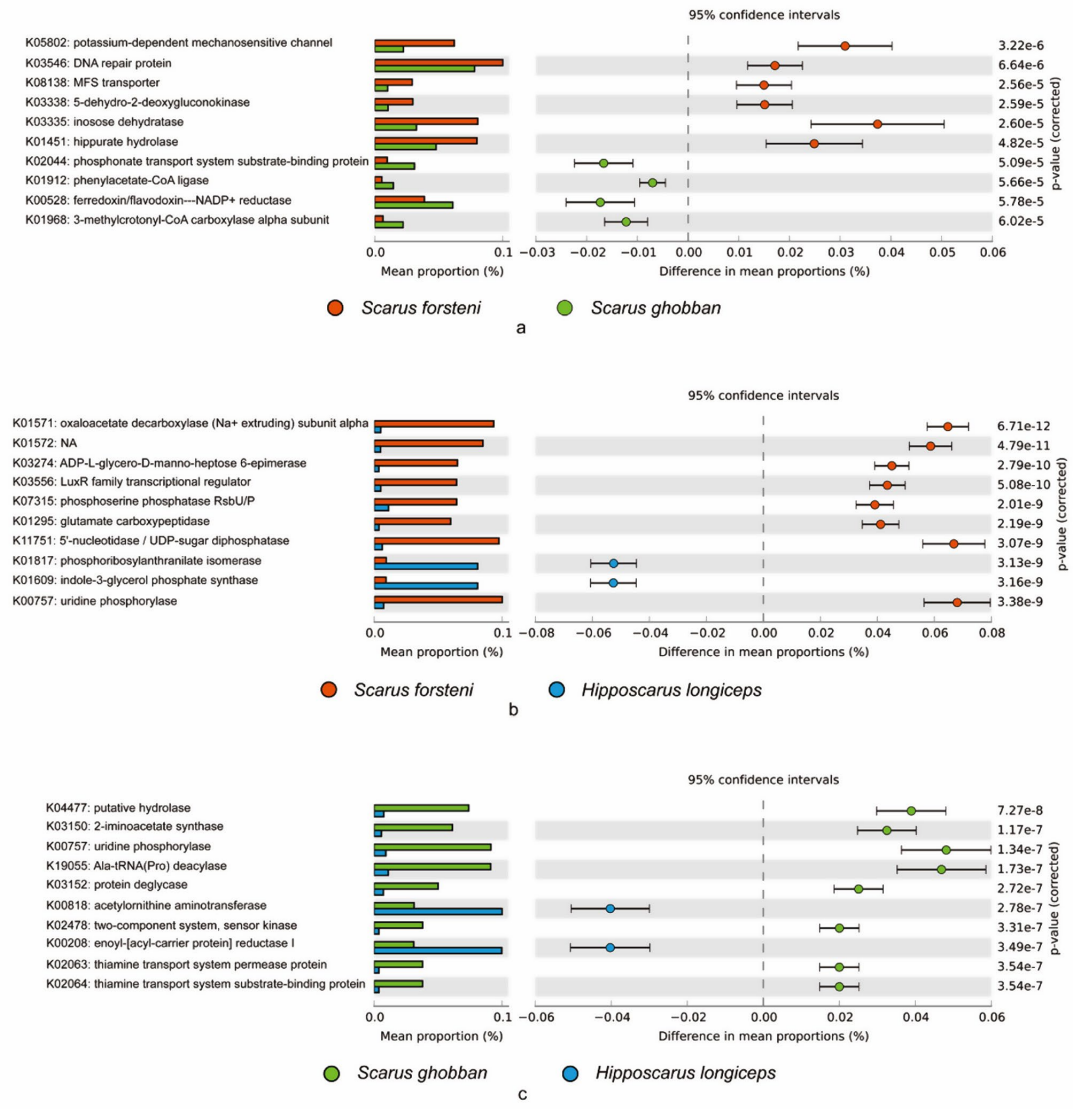
Top 10 differential function information between different species of parrotfish. **a**: *S. forsteni* vs. *S. ghobban*; **b**: *S. forsteni* vs. *H. longiceps*; **c**: *S. ghobban* vs. *H. longiceps*; The functional information was predicted by PICRUSt2 based on the indicator ASVs against KEGG database

**Fig. 9:**
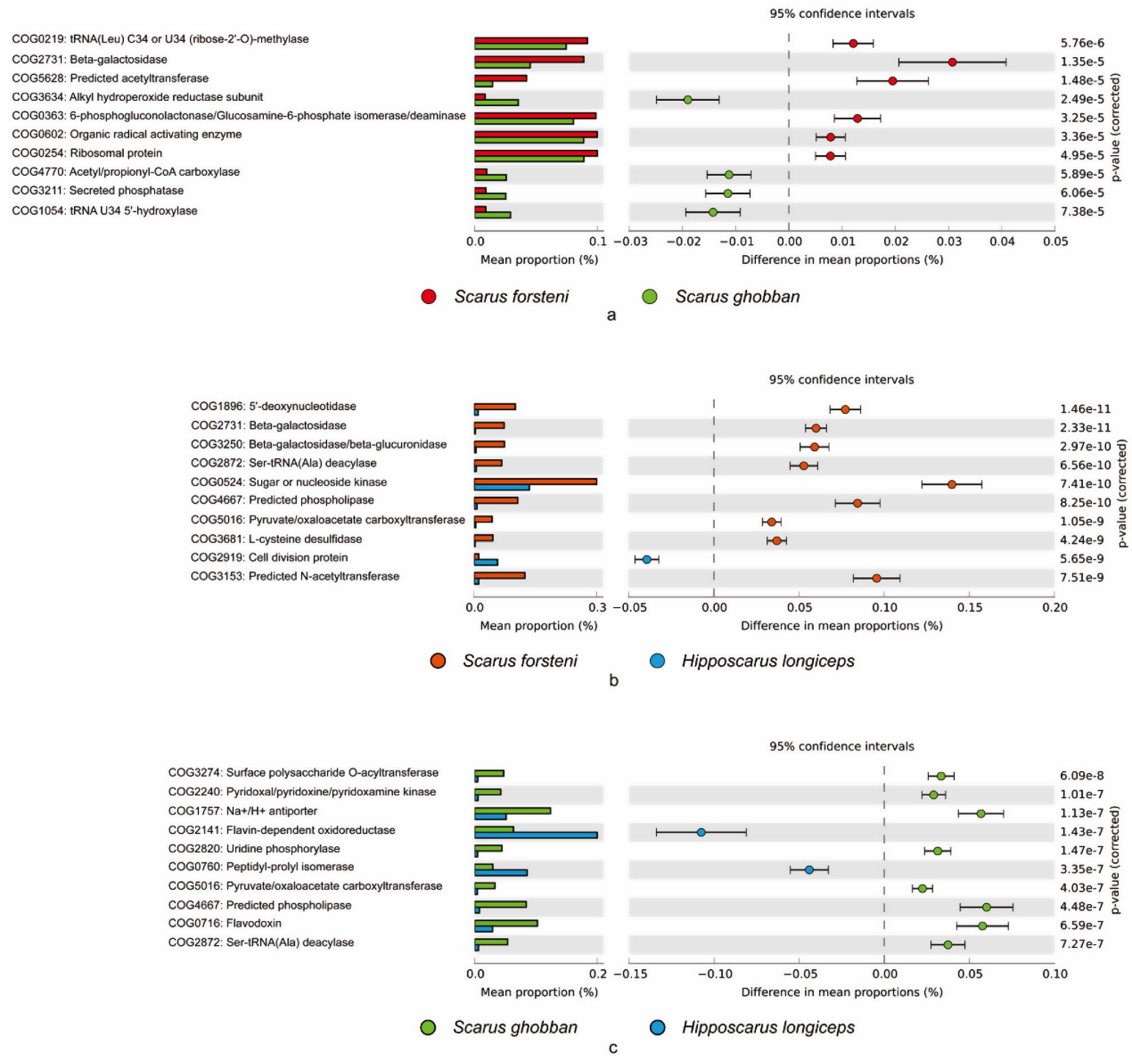
Top 10 differential function information between different species of parrotfish. **a**: *S. forsteni* vs. *S. ghobban*; **b**: *S. forsteni* vs. *H. longiceps*; **c**: *S. ghobban* vs. *H. longiceps*; The functional information was predicted by PICRUSt2 based on the indicator ASVs against COG database

Based on the KEGG database, the potassium-dependent mechanosensitive channel, DNA repair protein, MFS transporter, 5-dehydro-2-deoxygluconokinase, inosose dehydratase, hippurate hydrolase, were more abundant in the gut of *S. forsteni* than *S. ghobban* (Fig. 8a), oxaloacetate decarboxylase (Na + extruding) subunit alpha, ADP-L-glycero-D-manno-heptose 6-epimerase, LuxR family transcriptional regulator, phosphoserine phosphatase RsbU/P, glutamate carboxypeptidase, 5’-nucleotidase / UDP-sugar diphosphatase, and uridine phosphorylase were more dominant in the gut of *S. forsteni* than that of *H. longiceps* (Fig. 8b). Meanwhile putative hydrolase, 2-iminoacetate synthase, uridine phosphorylase, Ala-tRNA (Pro) deacylase, protein deglycase, two-component system (sensor kinase), thiamine transport system permease protein, and thiamine transport system substrate-binding protein were significantly enriched in the gut of *S. ghobban* compared to that of *H. longiceps* (Fig. 8c). Analysis based on the COG database showed that tRNA (Leu) C34 or U34 (ribose-2’-O)-methylase, beta-galactosidase, predicted acetyltransferase, 6-phosphogluconolactonase/Glucosamine-6-phosphate isomerase/deaminase, and ribosomal protein were more dominant in the gut microbes of *S. forsteni* than *S. ghobban* (Fig. 9). Meanwhile, compared to the gut of *H. longiceps*, the 5’-deoxynucleotidase, beta-galactosidase, beta-galactosidase/beta-glucuronidase, Ser-tRNA (Ala) deacylase, sugar or nucleoside kinase, predicted phospholipase, pyruvate/oxaloacetate carboxyltransferase, L-cysteine desulfidase, and predicted N-acetyltransferase were dominated in the gut of *S. forsteni* (Fig. 9b). In addition, surface polysaccharide O-acyltransferase, pyridoxal/pyridoxine/pyridoxamine kinase, Na+/H + antiporter, uridine phosphorylase, pyruvate/oxaloacetate carboxyltransferase, predicted phospholipase, flavodoxin, and Ser-tRNA (Ala) deacylase were significantly enriched in the gut of *S. ghobban* compared to that of *H. longiceps* (Fig. 9c).

## Discussion

The health of coral reefs is threatened by various disturbances that have led to a reduction in coral cover, loss of biodiversity and a reduction in ecosystem services [43, 59]. One of the main factors affecting the resilience of coral reefs is the proliferation of macroalgae, which inhibits the survival and growth of adult corals and the recruitment of juvenile corals. Macroalgae can also produce substances that trigger the growth of harmful microorganisms, leading to coral tissue degradation and disruption of the coral microbiome [43]. Parrotfish are a common sight on coral reefs and seagrass beds in the Indo-Pacific region and are important fishery targets, particularly in developing countries. These fish are crucial in maintaining the balance between corals and macroalgae, and their decline due to overfishing has been linked to the dominance of macroalgae [38, 60]. Although parrotfish are very common in coral reef areas, and are frequently used for ornamental and practical purposes, very little is known about the ecological role of their gut-associated microorganisms. According to a recent study by Chiarello et al. (2020), parrotfish microbiota may account for up to 2.5% of the total prokaryotic diversity on Earth, indicating that they represent a significant hotspot for microbial diversity [61].

In recent years, there has been extensive research on the gut microbes of parrotfish [43, 44, 62, 63]. These bacteria form complex communities in the intestinal tract that play crucial roles in development, immunity, health, protection against pathogens, and even behavior [64]. However, there have been relatively few comparative studies on the gut microbes of females and males after sex reversal in parrotfish. Although our results showed significant differences in the gut microbial community structure of the three species of parrotfish, *S. forsteni*, *S. ghobban* and *H. longiceps*, studied here, there were no significant differences in the gut microbial community structure of female parrotfish and males after sexual reversal. At the genus level, many of the OTUs were still considered unclassifiable taxa, while the classifiable ASVs were mainly composed of *Vibrio*, *Photobacterium*, *Enterococcus* and *Lactococcus*. Among them, *Vibrio* and *Photobacterium* were the dominant taxa in the gut of *S. forsteni*, which was consistent with the dominant microorganism in the gut of *C. sordidus* taken from the Palmyra Atoll coral reef [41], but while *Photobacterium* was also dominant in the gut of *S. ghobban*, the relative abundance of *Vibrio* was low. Researchers have isolated *Lactococcus* and *Enterococcus* from the guts of 12 species of fish, including fish of the family *Labridae*, and have confirmed that these bacteria have broad-spectrum antibacterial activity against some aquaculture pathogens such as *Vibrio* and *Photobacterium* [65]. Wrasse (Labridae) are vulnerable to Vibrio infection, including *Vibrio splendidus*, *Vibrio tapetis*, and *Vibrio anguillarum*. Yet al.though the health of wrasse is challenged by *Vibrio*, these vibrios appear to cause mortality only in some wrasse species [66–68].

In the marine environment, various bacteria use enzymes to degrade polysaccharides. For instance, the *Vibrio* isolated from the seawater of Mihonoseki harbor in Japan showed alginate depolymerization activity, and the deduced amino acid sequence of the alginate lyase gene of this marine strain showed a 92.3% homology with that of *Photobacterium* [69]. Microorganisms that affect their host organisms are particularly important, and some marine fish that feed on algae have microbes in their digestive tracts that produce carbohydrate hydrolytic enzymes, such as agarose and fucosidase. Researchers isolated 48 strains of *Vibrio* from the gut of algae-eating and fish that eat both algae and invertebrates, and found that the *Vibrio* present possessed many genes encoding alginate lyase and chitinase [70]. In addition, *Photobacterium* was also isolated from the gut of fish sampled from the coast of Shimane Peninsula, Japan. *Vibrio*, *Photobacterium*, and *Clostridium* are frequently reported as their dominant genera in the gut of marine fish, and a meta-analysis of marine fish gut communities showed that Vibrionales bacteria, including *Vibrio* and *Photobacterium*, accounted for 70% of the total sequence reads [71]. Studies have shown that fish gut microbiota may have a positive effect on the digestive process in fish, and these studies have isolated and identified a number of enzyme-producing microorganisms that promote digestion in fish, including *Bacillus*, *Vibrio*, *Photobacterium*, *Pseudomonas* and some lineages of *Enterobacteriaceae* [72]. Thus, a number of beneficial bacteria in the gut microbiome helps to maintain a healthy and balanced state of the host.

Sex reversal is a relatively common phenomenon in fish, and many cases are widely known. An in-depth understanding of the mechanisms of sex reversal in fish will help to understand the evolution and development of reproductive strategies in fish and the process of sex determination in vertebrates. Sex differences are pervasive in metabolic traits [73–75], and may arise from reversible hormonal influences, irreversible tissue developmental processes, and from differences in gene expression. Sex differences may depend on genetic background, environmental factors, and the gut microbiome, and it is now clear that the gut microbiota tends to differ between males and females, with potentially dramatic effects on disease susceptibility [76]. However, in our study, we did not find significant differences in the gut microbial community structure between the female parrotfish of *S. forsteni*, *S. ghobban* and *H. longiceps*, and the respective male parrotfish that were sexually reversed from females. Nevertheless, we observed significant differences in the gut microbial community structure between these three species of parrotfish. We identified indicator OTUs in the gut of each species and predicted the potential functions of these indicator OTUs to reveal functional differences in the gut microbes of these parrotfish. The gut microbes of *S. forsteni* showed superiority in aspartate and asparagine biosynthesis, histidine degradation, inositol degradation, heptose biosynthesis, chitin derivatives degradation, enterobactin biosynthesis, and thiazole biosynthesis. Asparagine can be derived from aspartate. Aspartate is an important substrate for nucleotide synthesis, and proliferating cells primarily require respiration for aspartate synthesis and aspartate-dependent nucleotide synthesis [77]. The pathway that produces formiminoglutamic acid (FIGLU) is the main metabolic pathway for histidine degradation and is catalyzed by histidase to deaminate to form urocanic acid, which is then converted to FIGLU [78]. Inositol is a chemical compound that has nine stereoisomers and is classified as a hexahydroxycyclohexane. Although it belongs to the vitamin complex, it is not considered a true nutrient because it can be synthesized by both prokaryotes and eukaryotes, as well as in the human body. However, it is known as a specific probiotic molecule and has a strong similarity to the glucose molecule [79–81]. ADP-L-glycero-β-D-manno-heptose (ADP-heptose), which is a soluble metabolite, is present during the lipopolysaccharide (LPS) synthesis pathway in different Gram-negative bacteria, and LPS is immunogenic, stimulating the production of specific antibodies by host B lymphocytes [82]. Chitin, a long-chain polymer of *N*-acetylglucosamine, is found in some unicellular organisms such as diatoms, protozoa and fungi, as well as in some multicellular organisms such as sponges, corals, mollusks, worms and arthropods, and acts as nutritional food source [83]. The ability of parrotfish to consume large amounts of food containing chitin is consistent with the significant involvement of its gut microbes in the degradation of this polysaccharide. Enterobactin biosynthesis belongs to siderophore and metallophore synthesis in secondary metabolite synthesis. Iron is an essential trace element. In some conditions, such as gut inflammation, the level of physiologically available iron is very low and becomes a limiting factor for bacterial growth. In order to survive, many bacteria have evolved specialized transport systems, called siderophores, which can retrieve iron ions. Enterobactin is the prototypic catecholate siderophore produced by many bacteria, such as *Escherichia*, *Marinobacte*r, and *Vibri* [84–86]. Moreover, although there are some gut microorganisms, such as some commensal Bacteroides, that do not produce siderophores, they can still grow using siderophores from other enterobacteria under iron-limiting conditions [87]. Vitamin B1, also known as thiamin diphosphate, plays a crucial role in energy metabolism that is essential for the nutrition of all living organisms. It is composed of two chemical compounds: pyrimidine, which is 4-amino-5-hydroxymethyl-2-methylpyrimidine, and thiazole, which is 5-(2-hydroxyethyl)-4-methylthiazole [88–91]. The biosynthetic pathway of thiazole is different between anaerobic prokaryotes, aerobic prokaryotes and eukaryotes [92]. In anaerobic bacteria, thiazole is derived from tyrosine [93, 94] and 1-deoxy-D-hydroxyglucose-5-phosphate [95], and several enzymes are involved in thiazole synthesis, whereas in aerobic bacteria, glycine is used instead of tyrosine [92]. However, even if the precursors are different, the enzymes involved in thiazole synthesis are similar in all prokaryotes [96].

## Conclusion

In birds and mammals, the sex determination system is conserved and stable, while in fish, one of the poikilotherms, it is highly variable. In fish as well as other poikilotherm vertebrates, sex reversal can be readily induced by hormones and sometimes by environmental factors. However, no one has compared the structure and composition of the gut microbial community of fish that underwent sex reversal with those of their own sex, and whether the gut microbial community structure would be significantly different or not. In our study, we compared the gut microbial community structure of female parrotfish with that of parrotfish reversed from female to male using the gut microbiomes from three species of parrotfish and found that there were no significant differences. We also predicted the function of the gut microbes and found no significant differences in potential function between females and males (sex-reversed). The gut microbes of these three species of parrotfish, *S. forsteni*, *S. ghobban* and *H. longicep*, have not been reported previously. Our study showed that the gut microorganisms of these parrotfish were mainly composed of *Vibrio*, *Photobacterium*, *Enterococcus* and *Lactococcus* at the genus level, *Vibrionaceae*, *Burkholderiaceae*, *Enterobacteriaceae*, *Streptococcacea*, and *Erwiniaceae* at the family level, and Pseudomonadota (class Gammaproteobacteria) and Bacillota at the phylum level. It is worth noting that although we have predicted the gut microbial functions of these parrotfish, this is likely to be slightly different from their real gut microbial functions. Meanwhile, although we have observed some functional differences, we have not been able to get to the cause of such differences, which may be related to the environment, differences in species, or different diets. In addition, 16 S amplicon sequencing results are susceptible to biases from different sources, such as the selection of variable regions and primers, differences in the quality of DNA templates, and the selection of sequencing platforms [97]. PICRUSt2 also has limitations, such as relying on pre-existing reference genomes, which may lead to less accurate functional predictions for some microorganisms in rare environments [48]. Therefore, future metagenomics, metatranscriptomic, metaproteomics or metabolomics work on the gut microbes of parrotfish is needed to fully understand the functional information of these organisms.

## Electronic Supplementary Material

Below is the link to the electronic supplementary material.

Supplementary Material 1

## Data Availability

The data that support the results are included within the article or available from the corresponding author on reasonable request. All raw sequencing reads were deposited in the NCBI database (http://www.ncbi.nlm.nih.gov/) under BioProject accession number: PRJNA970548 for the parrotfish gut microbial datasets.
