# Supplementary material for: Environmental sex reversal in parrotfish does not cause differences in the structure of their gut microbial communities
Source: BMC Microbiol. 2024 Dec 19;24:531. doi: 10.1186/s12866-024-03698-3 (PMC11657377; doi:10.1186/s12866-024-03698-3)
Supplement: Supplementary file 1 — Supplementary Material 1 [file 12866_2024_3698_MOESM1_ESM.docx]

**Supplemental figures and tables:**

**Supplemental figure S1.** Gut microbial community members of *S. forsteni*, *S. ghobban*, and *H. longiceps* at the family level. F refers to female samples and M refers to male following sex reversal samples.


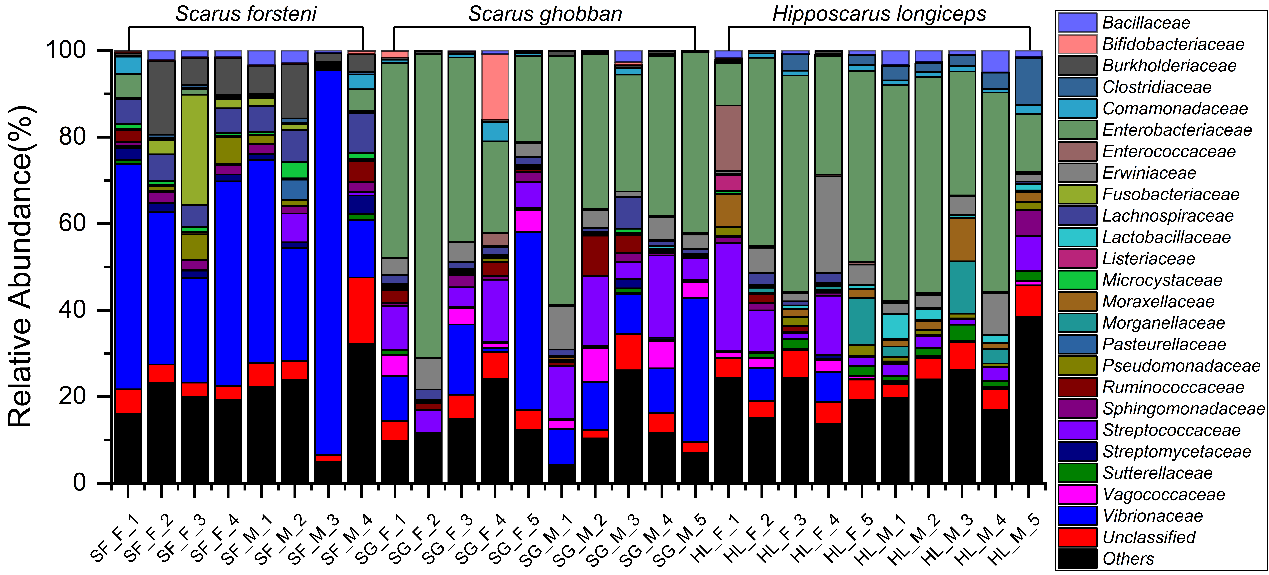


**Supplemental figure S2.** Gut microbial community members of *S. forsteni*, *S. ghobban*, and *H. longiceps* at the genus level. F refers to female samples and M refers to male following sex reversal samples.


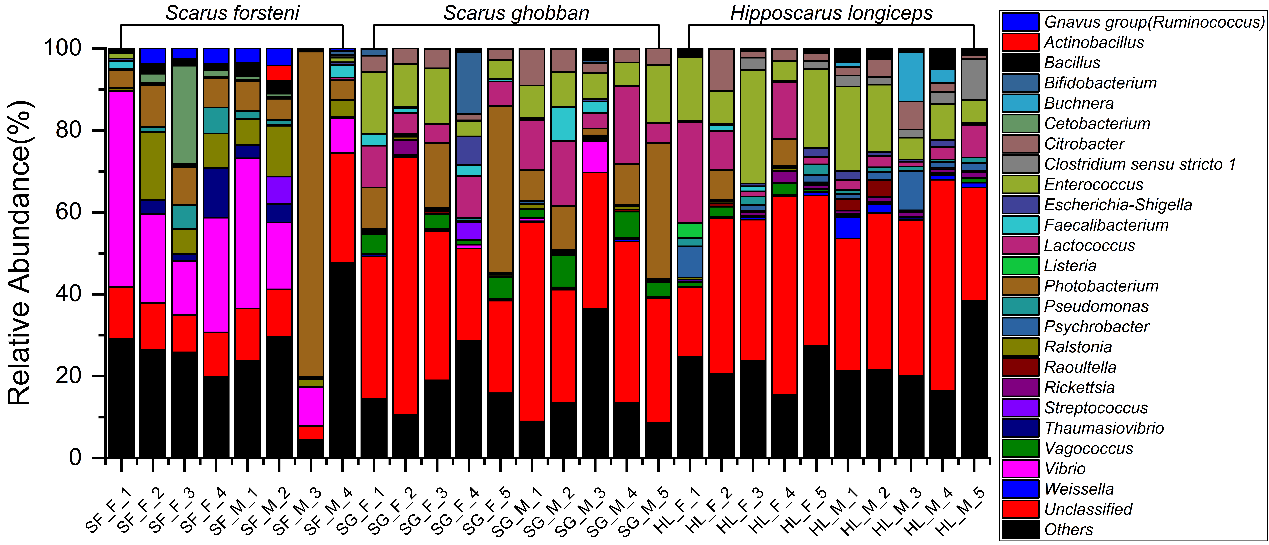


**Supplemental figure S3.** NMDS analysis of the gut microbial functional communities separated the samples into three principal groups, each group composed of gut samples from a single parrot fish species, *Scarus forsteni*, *Scarus ghobban*, and *Hipposcarus longiceps*, respectively. The results are based on the functional information predicted by PICRUSt2 using the MetaCyc database. The plots on the left (stress = 0.099) and right (stress = 0.075) were calculated using the Bray-Curtis dissimilarity index and the Jaccard similarity index, respectively.


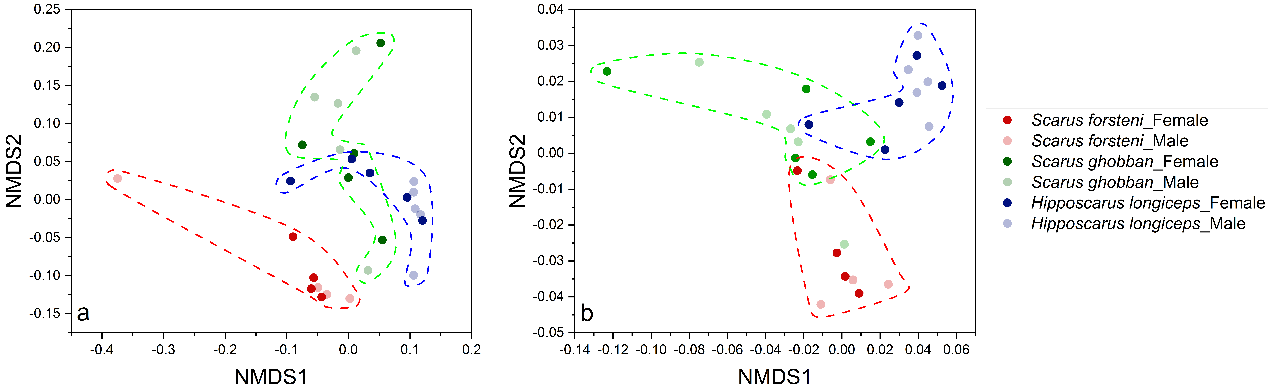


**Supplemental figure S4.** NMDS analysis of the gut microbial functional communities separated the samples into three principal groups, one composed of the gut samples of *Scarus forsteni*, a second group composed of gut samples from the *Scarus ghobban*, and a third group composed of gut samples from *Hipposcarus longiceps*. The results are based on the functional information predicted by PICRUSt2 using the COG database. The plots on the left (stress = 0.111) and right (stress = 0.077) were calculated using the Bray-Curtis dissimilarity index and the Jaccard similarity index, respectively.


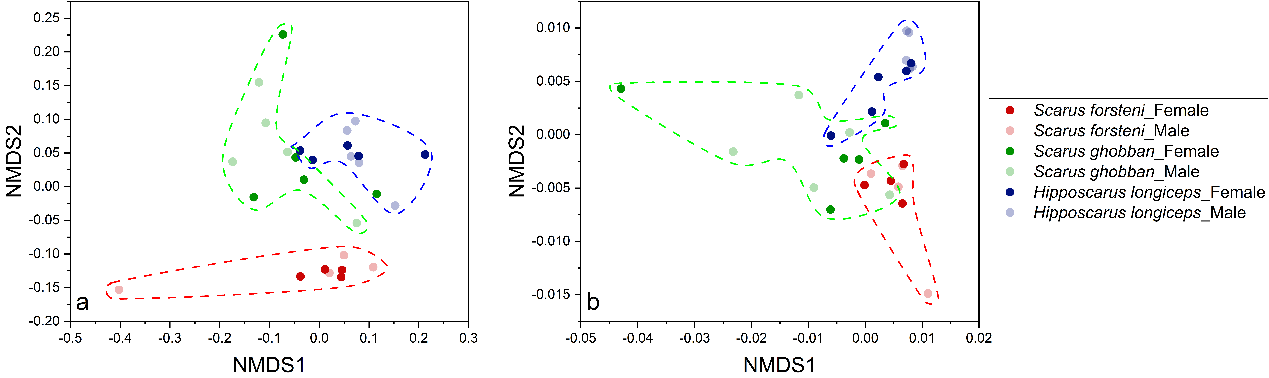


**Supplemental table S1.** Information of environmental factors about samples, such as the geographical location where the samples were collected and the temperature and salinity of the sea water.

| ***Hipposcarus longiceps*** | |  |  |  |  |
| --- | --- | --- | --- | --- | --- |
| **NO.** | Sample ID | Sex reversal | Location | Temperature | Salinity |
| YC20220507014 | YC014 | male | N16.30E111.30 | 26℃ | 3.40% |
| YC20220507010 | YC010 | male |  |  |  |
| YC20220507082 | YC082 | male |  |  |  |
| YC20220507050 | YC050 | male |  |  |  |
| YC20220507033 | YC033 | male |  |  |  |
| YC20220507053 | YC053 | female | N16.30E111.30 | 26℃ | 3.40% |
| YC20220507036 | YC036 | female |  |  |  |
| YC20220507008 | YC008 | female |  |  |  |
| YC20220507102 | YC102 | female |  |  |  |
| YC20220507045 | YC045 | female |  |  |  |
| ***Scarus ghobban*** |  |  |  |  |  |
| **NO.** | Sample ID | Sex reversal | Location | Temperature | Salinity |
| DQ20220514087 | DQ087 | female | N20.30E115.54 | 25℃ | 3.40% |
| DQ20220514078 | DQ078 | female |  |  |  |
| DQ20220514055 | DQ055 | female |  |  |  |
| DQ20220514098 | DQ098 | female |  |  |  |
| DQ20220514053 | DQ053 | female |  |  |  |
| DQ20220514058 | DQ058 | male | N20.30E115.54 | 25℃ | 3.40% |
| DQ20220514167 | DQ167 | male |  |  |  |
| DQ20220514075 | DQ075 | male |  |  |  |
| DQ20220514108 | DQ108 | male |  |  |  |
| DQ20220514054 | DQ054 | male |  |  |  |
| ***Scarus forsteni*** |  |  |  |  |  |
| **NO.** | Sample ID | Sex reversal | Location | Temperature | Salinity |
| DL20220428005 | DL005 | male | N16.40E112.40 | 30℃ | 3.34% |
| DL20220428001 | DL001 | male |  |  |  |
| DL20220428006 | DL006 | male |  |  |  |
| DL20220428004 | DL004 | male |  |  |  |
| DL20220428007 | DL007 | female | N16.40E112.40 | 30℃ | 3.34% |
| DL20220428009 | DL009 | female |  |  |  |
| DL20220428012 | DL012 | female |  |  |  |
| DL20220428010 | DL010 | female |  |  |  |

**Supplemental table S2. |** Tests of dissimilarity in the function of gut microbial communities of *Scarus forsteni*, *Scarus ghobban*, and *Hipposcarus longiceps* based on Bray-Curtis and Jaccard distances. The prediction of gut microbial community function was performed by PICRUSt2 based on the COG (Clusters of Orthologous Genes) database.

| Group | Bray-Curtis | Jaccard |
| --- | --- | --- |
|  | *p* | *p* |
| Group (*S. forsteni* and *S. ghobban*) | | |
| PERMANOVA | 0.001(**),  Pseudo-F = 7.108 | 0.003(**),  Pseudo-F = 4.604 |
| MRPP | 0.001(**),  Delta = 0.178 | 0.002(**),  Delta = 0.012 |
| ANOSIM | 0.002(**),  r = 0.371 | 0.014(*),  r = 0.202 |
| Group (*S. forsteni* and *H. longiceps*) | | |
| PERMANOVA | 0.002(**),  Pseudo-F = 10.757 | 0.001(**),  Pseudo-F = 11.051 |
| MRPP | 0.001(**),  Delta = 0.150 | 0.001(**),  Delta = 0.008 |
| ANOSIM | 0.001(**),  r = 0.600 | 0.001(**),  r = 0.664 |
| Group (*S. ghobban* and *H. longiceps*) | | |
| PERMANOVA | 0.096,  Pseudo-F = 2.021 | 0.002(**),  Pseudo-F = 8.206 |
| MRPP | 0.027(*),  Delta = 0.169 | 0.001(**),  Delta = 0.011 |
| ANOSIM | 0.025(*),  r = 0.127 | 0.001(**),  r = 0.466 |

* Difference is significant at 0.05 level, ** difference is significant at 0.01 level. *S. forsteni* refer to the Forsten's parrotfish *Scarus forsteni*; *S. ghobban* refer to the blue-barred parrotfish *Scarus ghobban*; and *H. longiceps* refer to the pacific longnose parrotfish *Hipposcarus longiceps*.

**Supplemental table S3 |** Tests of dissimilarity in the function of gut microbial communities of *Scarus forsteni*, *Scarus ghobban*, and *Hipposcarus longiceps* based on Bray-Curtis and Jaccard distances. The prediction of gut microbial community function was performed by PICRUSt2 based on the COG (Clusters of Orthologous Genes) database.

| Group | Bray-Curtis | Jaccard |
| --- | --- | --- |
|  | *p* | *p* |
| *S. forsteni* (Females and Males after sexual reversal) | | |
| PERMANOVA | 1,  Pseudo-F = 0.592 | 0.830,  Pseudo-F = 0.595 |
| MRPP | 0.925,  Delta = 0.163 | 0.711,  Delta = 0.009 |
| ANOSIM | 0.602,  r = -0.042 | 0.594,  r = -0.036 |
| *S. ghobban* (Females and Males after sexual reversal) | | |
| PERMANOVA | 0.917,  Pseudo-F = 0.222 | 0.922,  Pseudo-F = 0.469 |
| MRPP | 0.881,  Delta = 0.204 | 0.841,  Delta = 0.015 |
| ANOSIM | 0.865,  r = -0.116 | 0.909,  r = -0.010 |
| *H. longiceps* (Females and Males after sexual reversal) | | |
| PERMANOVA | 0.507,  Pseudo-F = 0.858 | 0.006(**),  Pseudo-F = 2.936 |
| MRPP | 0.401,  Delta = 0.144 | 0.022(*),  Delta = 0.007 |
| ANOSIM | 0.502,  r = -0.020 | 0.034(*),  r = 0.204 |
| All (Females and Males after sexual reversal) | | |
| PERMANOVA | 0.949,  Pseudo-F = 0.259 | 0.833,  Pseudo-F = 0.497 |
| MRPP | 0.978,  Delta = 0.205 | 0.754,  Delta = 0.014 |
| ANOSIM | 0.983,  r = -0.057 | 0.575,  r = -0.017 |

* Difference is significant at 0.05 level, ** difference is significant at 0.01 level. *S. forsteni* refer to the Forsten's parrotfish *Scarus forsteni*; *S. ghobban* refer to the blue-barred parrotfish *Scarus ghobban*; and *H. longiceps* refer to the pacific longnose parrotfish *Hipposcarus longiceps*.

**Supplemental table S4 |** Tests of dissimilarity in the function of gut microbial communities of *Scarus forsteni*, *Scarus ghobban*, and *Hipposcarus longiceps* based on Bray-Curtis and Jaccard distances. The prediction of gut microbial community function was performed by PICRUSt2 based on the MetaCyc database.

| Group | Bray-Curtis | Jaccard |
| --- | --- | --- |
|  | *p* | *p* |
| Group (*S. forsteni* and *S. ghobban*) | | |
| PERMANOVA | 0.001(**),  Pseudo-F = 13.480 | 0.004(**),  Pseudo-F = 6.629 |
| MRPP | 0.001(**),  Delta = 0.140 | 0.003(**),  Delta = 0.027 |
| ANOSIM | 0.001(**),  r = 0.505 | 0.009(**),  r = 0.264 |
| Group (*S. forsteni* and *H. longiceps*) | | |
| PERMANOVA | 0.001(**),  Pseudo-F = 21.471 | 0.001(**),  Pseudo-F = 17.978 |
| MRPP | 0.001(**),  Delta = 0.111 | 0.002(**),  Delta = 0.022 |
| ANOSIM | 0.001(**),  r = 0.758 | 0.001(**),  r = 0.818 |
| Group (*S. ghobban* and *H. longiceps*) | | |
| PERMANOVA | 0.221,  Pseudo-F = 1.464 | 0.001(**),  Pseudo-F = 15.388 |
| MRPP | 0.061,  Delta = 0.139 | 0.001(**),  Delta = 0.266 |
| ANOSIM | 0.063,  r = 0.090 | 0.001(**),  r = 0.589 |

* Difference is significant at 0.05 level, ** difference is significant at 0.01 level. *S. forsteni* refer to the Forsten's parrotfish *Scarus forsteni*; *S. ghobban* refer to the blue-barred parrotfish *Scarus ghobban*; and *H. longiceps* refer to the pacific longnose parrotfish *Hipposcarus longiceps*.

**Supplemental table S5 |** Tests of dissimilarity in the function of gut microbial communities of *Scarus forsteni*, *Scarus ghobban*, and *Hipposcarus longiceps* based on Bray-Curtis and Jaccard distances. The prediction of gut microbial community function was performed by PICRUSt2 based on the MetaCyc database.

| Group | Bray-Curtis | Jaccard |
| --- | --- | --- |
|  | *p* | *p* |
| *S. forsteni* (Females and Males after sexual reversal) | | |
| PERMANOVA | 0.945,  Pseudo-F = 0.499 | 0.727,  Pseudo-F = 0.574 |
| MRPP | 0.919,  Delta = 0.113 | 0.606,  Delta = 0.023 |
| ANOSIM | 0.682,  r = -0.042 | 0.817,  r = -0.120 |
| *S. ghobban* (Females and Males after sexual reversal) | | |
| PERMANOVA | 0.916,  Pseudo-F = 0.203 | 0.578,  Pseudo-F = 0.656 |
| MRPP | 0.912,  Delta = 0.172 | 0.557,  Delta = 0.032 |
| ANOSIM | 0.886,  r = -0.104 | 0.457,  r = -0.014 |
| *H. longiceps* (Females and Males after sexual reversal) | | |
| PERMANOVA | 0.443,  Pseudo-F = 0.844 | 0.076,  Pseudo-F = 1.770 |
| MRPP | 0.423,  Delta = 0.114 | 0.075,  Delta = 0.021 |
| ANOSIM | 0.572,  r = -0.056 | 0.124,  r = 0.110 |
| All (Females and Males after sexual reversal) | | |
| PERMANOVA | 0.959,  Pseudo-F = 0.158 | 0.769,  Pseudo-F = 0.401 |
| MRPP | 0.977,  Delta = 0.178 | 0.748,  Delta = 0.037 |
| ANOSIM | 0.984,  r = -0.053 | 0.762,  r = -0.035 |

* Difference is significant at 0.05 level, ** difference is significant at 0.01 level. *S. forsteni* refer to the Forsten's parrotfish *Scarus forsteni*; *S. ghobban* refer to the blue-barred parrotfish *Scarus ghobban*; and *H. longiceps* refer to the pacific longnose parrotfish *Hipposcarus longiceps*.
